# Study on the Spatial and Temporal Distribution Characteristics and Influencing Factors of Particulate Matter Pollution in Coal Production Cities in China

**DOI:** 10.3390/ijerph19063228

**Published:** 2022-03-09

**Authors:** Ju Wang, Tongnan Li, Zhuoqiong Li, Chunsheng Fang

**Affiliations:** College of New Energy and Environment, Jilin University, Changchun 130012, China; litn20@mails.jlu.edu.cn (T.L.); zhuoqiong21@mails.jlu.edu.cn (Z.L.); fangcs@jlu.edu.cn (C.F.)

**Keywords:** air pollution, PM_2.5_, PM_10_, coal production city, PSCF, socio-economic factors

## Abstract

In recent years, with the continuous advancement of China’s urbanization process, regional atmospheric environmental problems have become increasingly prominent. We selected 12 cities as study areas to explore the spatial and temporal distribution characteristics of atmospheric particulate matter in the region, and analyzed the impact of socioeconomic and natural factors on local particulate matter levels. In terms of time variation, the particulate matter in the study area showed an annual change trend of first rising and then falling, a monthly change trend of “U” shape, and an hourly change trend of double-peak and double-valley distribution. Spatially, the concentration of particulate matter in the central and southern cities of the study area is higher, while the pollution in the western region is lighter. In terms of social economy, PM_2.5_ showed an “inverted U-shaped” quadratic polynomial relationship with Second Industry and Population Density, while it showed a U-shaped relationship with Generating Capacity and Coal Output. The results of correlation analysis showed that PM_2.5_ and PM_10_ were significantly positively correlated with NO_2_, SO_2_, CO and air pressure, and significantly negatively correlated with O_3_ and air temperature. Wind speed was significantly negatively correlated with PM_2.5_, and significantly positively correlated with PM_10_. In terms of pollution transmission, the southwest area of Taiyuan City is a high potential pollution source area of fine particles, and the long-distance transport of PM_2.5_ in Xinjiang from the northwest also has a certain contribution to the pollution of fine particles. This study is helpful for us to understand the characteristics and influencing factors of particulate matter pollution in coal production cities.

## 1. Introduction

In recent years, China’s rapid economic development and substantial growth in energy consumption have led to serious urban air pollution, especially particulate pollution [1,2]. Particulate matter pollution will affect air quality [3] and visibility [4] within the region, and endanger human health [5]. Due to the continuous fermentation of haze weather in China, PM_2.5_ pollution has attracted widespread attention in academic circles. At present, particulate matter has become the research focus of many scholars, mainly including pollution characteristic analysis [6], influencing factor analysis [7], pollution transmission analysis [8], health assessment [9], source analysis [10], air quality simulation [11] and so on. Most of these studies focus on economically developed regions such as the Beijing–Tianjin–Hebei [12], Yangtze River Delta [13] and Pearl River Delta [14], while there are fewer studies on the central and western regions of China and cities in functional areas.

The process of coal mining will not only significantly increase the prevalence of diseases such as cancer [15] and respiratory diseases [16] among coal workers, but also have varying degrees of health impact on the general population near coal mining [17,18,19]. According to Javier Cortes-Ramirez [20] et al., a review of 28 epidemiological studies found evidence that coal mining is associated with a variety of diseases in the population surrounding mining activities, particularly cancer and congenital anomalies. Teklit Zerizghi [21] et al. quantified the ecological and human health risks of heavy metal pollution in the soil around coal mining areas and found that the heavy metal content exceeds the local background concentration, and Cr is the main ecological and human health risk factor metal in the region. More studies [22,23] pointed out that children are more susceptible than adults to the health risks of heavy metals in particulate matter. The use of coal for household consumption [24,25] also increases human health risks.

In addition, the economy in central China is underdeveloped, mainly in Inner Mongolia, Shanxi and Shaanxi, with primary energy mining and deep processing as the main industry. Coal occupies a dominant position in China’s energy structure, and its mining, processing and transformation process can easily cause environmental pollution [26] and ecological degradation. Under the unique economic structure and geographical environment, the pollution characteristics of these regions are different from other regions.

This study selected 12 cities located in Shanxi Province, Inner Mongolia Autonomous Region and Shaanxi Province as the research area to explore the spatial and temporal distribution characteristics of atmospheric particulate matter in this region, and analyzed the influence of socioeconomic factors and natural factors on the local particulate matter level. This paper aims to study the temporal and spatial distribution characteristics and influencing factors of atmospheric particulate matter in the main coal production areas, so as to have a deeper understanding of the air pollution processes and influencing factors in this area.

## 2. Materials and Methods

### 2.1. Research Area and Data Sources

**Figure 1:**
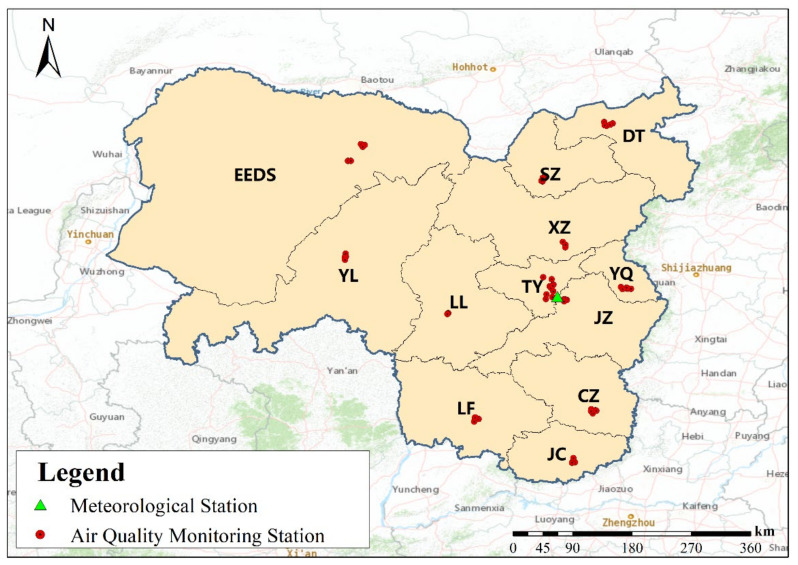
Air quality monitoring stations and the meteorological station in major coal production areas.

This study selected 12 coal cities in Shanxi Province, Inner Mongolia Autonomous Region and Shaanxi Province as the research objects, including ten cities in Shanxi Province (Datong(DT), Yangquan(YQ), Shuozhou(SZ), Xinzhou(XZ), Linfen(LF), Taiyuan(TY), Jinzhong(JZ), Jincheng(JC), Lvliang(LL) and Changzhi(CZ)), Ordos (EEDS) in Inner Mongolia Autonomous Region and Yulin (YL) in Shaanxi Province. In this study, the hourly concentration data of pollutants from 1 January 2015 to 31 December 2019 of 64 air quality monitoring stations in the study area and the meteorological monitoring data of Taiyuan meteorological station in 2019 were collected (Figure 1). The data sources are https://www.aqistudy.cn/historydata/ (accessed on 21 September 2021) and http://data.cma.cn (accessed on 25 September 2021), respectively. The pollutant data includes six pollutants: PM_2.5_, PM_10_, Carbon Monoxide (CO), Sulfur Dioxide (SO_2_), Ozone (O_3_) and Nitrogen Dioxide (NO_2_), and the meteorological data includes air temperature (T), air pressure (P), wind speed (WS) and wind direction (WD). According to the “Ambient Air Quality Standard” (GB 3095-2012), the missing values in the data are processed to improve the accuracy of the monitoring data. In calculating the average daily concentration, we require at least 20 h of average concentration or sampling time, otherwise, the average daily concentration is considered invalid. When calculating the monthly average concentration, we require at least 27 (February: 25) daily average concentration values, otherwise, the monthly average concentration is considered invalid. When calculating the annual average concentration, we require at least 324 daily average concentrations, otherwise, the annual average concentration is considered invalid.

### 2.2. Analysis of Socioeconomic Factors

The potential impact of socioeconomic indicators on particulate pollution has been widely discussed. On the basis of existing studies [27], we selected five indicators, including gross domestic product(GDP) [28,29], population density(PD), and secondary industry(SI) [30,31], coal output(CO) and generating capacity(GC). The annual statistics of GDP, PD, SI, CO and GC come from the statistical yearbooks of each city. Table S1 provides detailed information on these socioeconomic factors for each city. In order to determine the association between particulate matter concentrations and socioeconomic factors, the annual average concentration data of PM_2.5_ and PM_10_ were used as dependent variables, and socioeconomic factors were used as independent variables. The curve fitting analysis is carried out in the form of scatter plots and curve graphs to illustrate the relationship between variables. According to the determination coefficient (R^2^) and significance test (*p* < 0.05), the fitting curve was used to characterize the impact of socioeconomic factors on particulate pollution.

### 2.3. Trajectory Analysis

#### 2.3.1. Cluster Analysis

In this study, the HYSPLIT [32] model (http://ready.arl.noaa.gov/HYSPLIT.php)( accessed on 15 December 2021) was used to calculate the 72-h backward trajectory at a height of 500 m in Taiyuan city from 2015 to 2019. The time interval was 1 h, and 8760 or 8784 trajectories can be obtained every year. Using the Euclidean distance clustering algorithm in TrajStat software [33], the air mass trajectories arriving in Taiyuan were clustered.

#### 2.3.2. Potential Source Contribution Function (PSCF)

Based on the HYSPLIT [32] model provided by the National Oceanic and Atmospheric Administration (NOAA) Air Resources Laboratory and the Australian Bureau of Meteorology, the grid analysis was carried out on the area where the study area was located, and the grid spacing was 0.25° × 0.25°. By combining the trajectory of the air mass and the value of a certain element [34], PSCF gives possible emission source locations, and can preliminarily determine the impact of long-distance migration of pollutants on the study area. PSCF is defined as Equation (1).

(1) $$\mathrm{PSCF}=\frac{m_{ij}}{n_{ij}}$$

Since PSCF [35,36] is developed based on conditional probability functions, the error of PSCF increases as the distance between the grid and the sampling point increases. For some grids with fewer trajectories, the calculated PSCF values have large uncertainties. In order to reduce the uncertainty of PSCF, the weight function W_ij_ [37] needs to be introduced. The W_ij_ settings determined this time are as follows:

(2) $$W_{i}=\left\{\;\begin{array}{c} 1.0\;\;\;\;\;\;\;\;\;\;\;\;\;\;\;\;\;n_{i}>80\;\\ \;0.7\;\;\;\;\;\;\;\;80\geq n_{i}>20\\ 0.42\;\;\;\;\;\;20\geq n_{i}>10\\ 0.05\;\;\;\;\;\;\;\;10\geq n_{i}>0 \end{array}\right.$$

## 3. Results and Discussion

### 3.1. Spatial–Temporal Distribution Characteristics of Particulate Matter Concentration

**Figure 2:**
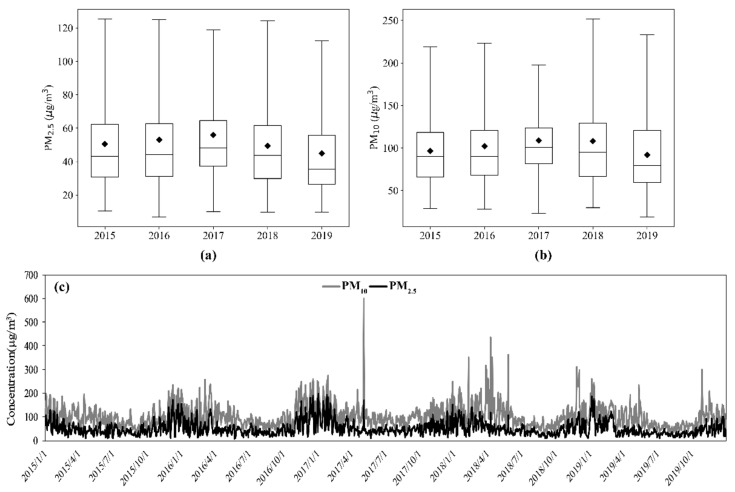
Temporal variation of PM concentrations in the study area from 2015 to 2019: (**a**) Annual variation of PM_2.5_; (**b**) annual variation of PM_10_; (**c**) five-year time series of PM_2.5_ and PM_10_.

Figure 2 shows the annual change of the mass concentration of particulate matter in the study area from 2015 to 2019. On the whole, the annual average concentration of PM_2.5_ and PM_10_ increased first and then decreased, showing an inverted U-shaped distribution, reaching the peak in 2017. It can be seen from Figure S1 that EEDS, YL, DT and XZ show a trend of first decreasing, then increasing and then decreasing. The rest of the cities showed a trend of first increasing and then decreasing, with the maximum value appearing in 2016 or 2017. Among the 12 cities, the maximum annual PM_2.5_ concentration was 81.93 μg/m^3^ occurring in LF in 2017. In the past five years, the average annual PM_2.5_ concentration of LF city increased slightly, and of other cities decreased to varying degrees. Among them, XZ had the most decrease, from 58.67 μg/m^3^ in 2015 to 42.46 μg/m^3^ in 2019, dropping 27.6%; however, the annual average PM_2.5_ concentration in EEDS and YL had little change, the concentration curve was relatively gentle and the PM_2.5_ pollution was relatively light.

As can be seen from Figure 2b, the variation trend of PM_10_ is similar to PM_2.5_ [38,39], with the highest concentration in 2017. As shown in Figure S1, except YQ and CZ, PM_10_ concentrations in other cities all reached their highest values in 2017 or 2018. The maximum PM_10_ concentration was 132.04 μg/m^3^ occurring in TY in 2018. Different from the pattern of PM_2.5_ above, there were five cities in the region where PM_10_ concentration increased in 2019 compared with 2015, with the highest increase of 14.7% in SZ. PM_10_ concentration in other cities had decreased, and YQ had the most obvious decrease, from 113.72 μg/m^3^ in 2015 to 88.30 μg/m^3^ in 2019, with a decrease of 22.4%. In terms of the whole study area, the annual average concentration of PM_2.5_ and PM_10_ in 2019 decreased significantly compared with 2015, with 44.98 μg/m^3^ and 91.76μg/m^3^, respectively, but both exceeded the secondary standard limit of Ambient Air Quality Standard (GB3095-2012).

The five-year time series of PM_2.5_ and PM_10_ in the study area are shown in Figure 2c. On the whole, PM_2.5_ presented a distribution characteristic of low concentration in summer and high concentration in winter. The monthly variation of PM_2.5_ shows a significant U-shaped change pattern, reaching the highest in January and the lowest in August. Conditions such as low temperature, relatively stable atmosphere structure and a large number of pollutants emitted by coal-fired heating [39] lead to the high concentration of particulate matter in winter. Precipitation can significantly reduce ambient PM_2.5_ concentrations in summer. However, the concentration of PM_10_ showed many peaks from March to May in 2017, 2018 and 2019, which was due to the occurrence of several serious typical strong sandstorm events in this period, and the contribution of coarse particles to PM_10_ in this region increased significantly [40]. Among them, the concentration of PM_10_ on 4 May 2017, reached the highest, which was 599.95 μg/m^3^.

**Figure 3:**
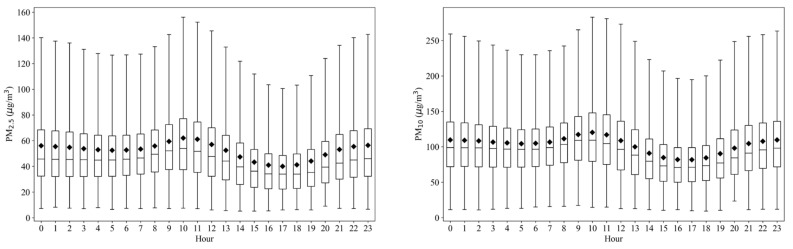
Diurnal variation of PM_2.5_ and PM_10_ concentrations in the study area during 2015–2019.

Figure 3 shows the diurnal variation of PM_2.5_ and PM_10_ concentrations during 2015–2019 in the study area. The variation trend of PM_2.5_ and PM_10_ concentrations in the study area is basically the same, showing an obvious double peak and double valley distribution. The first peak appeared at 10:00–11:00, and the second at 22:00–23:00, which is smaller than the first peak. The reason for traffic rush pollution in the morning is the enhancement of human activities [41], and a large amount of tail gas is emitted by vehicles. After the photochemical reaction, secondary aerosols are generated, which makes PM_2.5_ concentration reach the first peak. With the diffusion of pollutants, the concentration gradually decreases and reaches a valley at 17:00 [42]. With the arrival of the evening traffic peak [43] and the decrease in night temperature, it is easy to form a temperature inversion, and the atmosphere is relatively stable, which is not conducive to the diffusion of pollutants, causing the PM_2.5_ concentration to increase again at night and reach its peak again.

**Figure 4:**
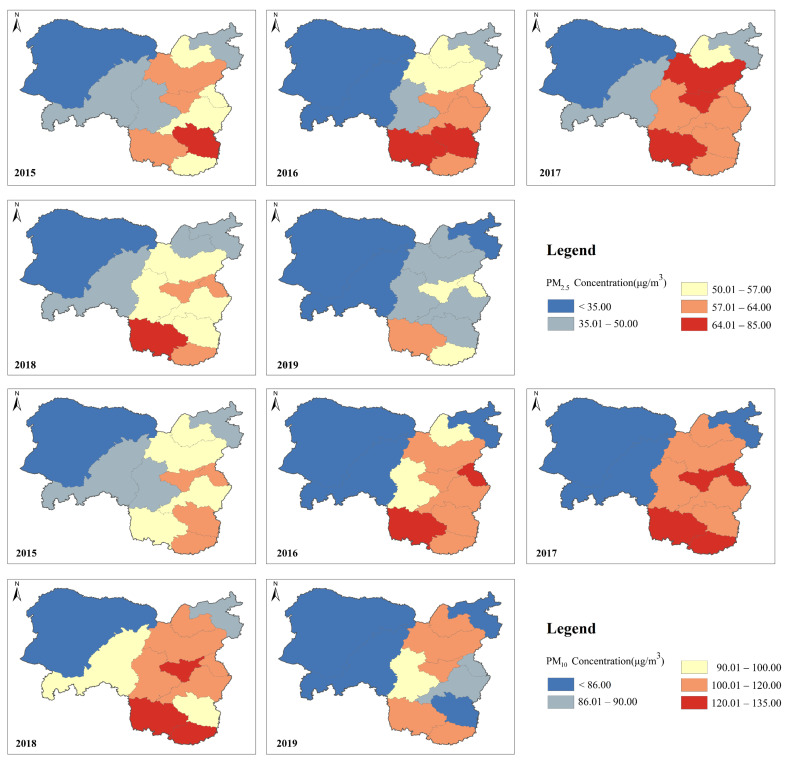
Spatial distribution of PM_2.5_ and PM_10_ in the study area from 2015 to 2019.

To further understand the characteristics of particulate matter in coal cities, we made statistics of the annual average concentrations of PM_2.5_ and PM_10_ in each city from 2015 to 2019, as shown in Figure 4. It can be found that the average annual concentration of PM_2.5_ and PM_10_ in the study area increased first and then decreased.

The severe PM_2.5_ pollution in 2015 occurred in CZ located in the southeast of the study area, with a concentration of 64.18 μg/m^3^. In 2016 and 2017, the annual average PM_2.5_ concentration in XZ, TY and LL increased significantly. The pollution scope was further expanded, and the pollution level in the eastern part of the study area was significantly aggravated. From 2018 to 2019, PM_2.5_ pollution in the study area as a whole was effectively alleviated, and the annual average PM_2.5_ concentration in each city decreased, indicating that good results have been achieved in the prevention and control of air pollution in the past two years.

Similar to the pattern of PM_2.5_, the severe pollution area of PM_10_ is still concentrated in the eastern part of the study area. PM_10_ pollution levels were the most serious in 2017, and YQ, TY, JC and LF have the highest PM_10_ concentration. In 2019, the annual average concentration of PM_10_ decreased significantly. Some cities located in the southeast and central parts of the study area had relatively high concentrations of PM_2.5_ and PM_10_. During the five years, EEDS and YL, which are located in the western part of the study area, had low concentrations of PM_2.5_ and PM_10_, and the overall pollution level was low.

### 3.2. Influence of Social-Economic Factors on Particulate Matter

The study area is located in the central region of China, with slow social-economic development. Compared with the developed eastern region, there is a different correlation between social-economic factors and particulate matter pollution. In order to further explore the influence of social-economic factors on particulate matter in the study area, we conducted statistics on social-economic factors from 2015 to 2019, as shown in Figure S2. Compared with other cities, SI and GDP of EEDS, YL and TY are higher. TY has a higher PD; EEDS and YL have higher GC; EEDS, SZ and YL have higher CO. There is a great difference in the distribution of social-economic factors in different cities. From 2015 to 2019, the regional differences of socioeconomic factors in each year did not change much.

Figures S3–S5 show the fitting curve of particulate matter concentration and social-economic factors from 2015 to 2019. The parameters of the curve are shown in Table 1. PM_2.5_ was significantly correlated with SI, PD, GC and CO, while PM_10_ was only significantly correlated with GC and CO, which had a poor correlation with other socioeconomic factors. This may be because the sources of the two particles are different. Fine particles are greatly affected by human factors, while coarse particles are greatly affected by surrounding dust [44]; however, coal mining will have a negative impact on land cover and make a certain contribution to local particulate matter levels [45].

It is worth noting that PM_2.5_ presents an “inverted U-shaped” quadratic polynomial relationship with SI and PD, while presenting a U-shaped relationship with GC and CO. However, the correlation between particulate matter concentration and traditional social-economic factor GDP is not significant, because TY’s GDP is higher, but particulate matter pollution is heavier, showing a deviation from the overall relationship. Some studies [27] reported a positive correlation between SI and PM_2.5_, which was different from our results. This may be due to the large difference in economic development between cities in the study area, and those cities with higher industrial output value also invest more in environmental governance. Likewise, the reason why the cities with high CO express a negative relationship is that those have a complete system of coal mining and transformation in situ [46,47], while coal enterprises in the cities with low CO often use the basic pattern to mining due to the limits of its small-scale, which easily causes environmental degradation. Thereby, there is a negative relationship between PM_2.5_ and GC, CO.

This spatial correlation expresses the influence of local economic development and industrial pattern on local particulate matter level, which can provide a reference for the balance between regional economic development and environmental quality. There are still many shortcomings in this study, and the effects of various industries on particulate pollution can be refined in the follow-up work. In addition to human factors, particulate levels are also affected by natural factors such as meteorology and atmosphere transport.

### 3.3. Influence of Natural Factors on Particulate Matter

Through the calculation of the pollution centroid [48], we determine TY as the particle pollution centroid city in the study area. At the same time, in order to explain the unique particulates pollution characteristics of TY, we took it as an example to discuss the influence of natural factors on the concentration of the local particulate.

We calculated Pearson correlation coefficients between six conventional pollutants and three meteorological factors (Temperature (T), Pressure (P) and Wind Speed (WS)) in TY in 2019 to discuss their influence on the particulate concentration, and the results are shown in Table 2. PM_2.5_ and PM_10_ are significantly positively correlated with NO_2_, SO_2_, CO and air pressure, and significantly negatively correlated with O_3_ and Temperature. This is because NO_2_ and SO_2_ in the atmosphere can be converted into secondary inorganic aerosols, thus promoting the formation of particulate matter to a certain extent; the atmospheric stability is higher under high pressure and low temperature, which is not conducive to the diffusion of particulate matter. It is worth noting that wind speed has a significant negative correlation with PM_2.5_ and a significant positive correlation with PM_10_. Higher wind speed is conducive to the diffusion of fine particles [49,50], but it is easy to produce dust weather, which increases the concentration of PM_10_ in the atmosphere, indicating that there are certain differences between the sources of PM_2.5_ and PM_10_. In addition, the significant correlation between particulate matter and CO, which is commonly used to indicate fossil fuel combustion, suggests that particulate pollution is influenced by local source emissions.

**Figure 5:**
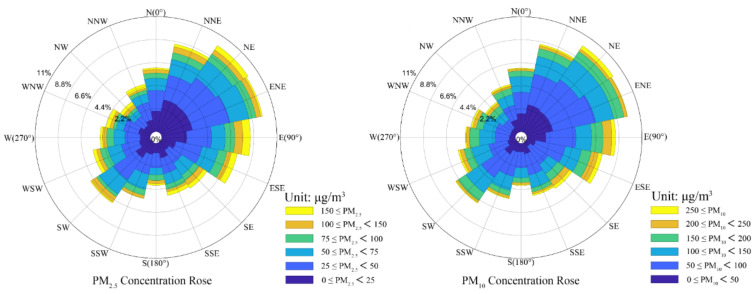
Pollutant rose diagram of wind direction and PM concentration.

Figure 5 is the pollutant rose diagram of wind direction and PM concentration. It can be seen that the PM_10_ and PM_2.5_ concentration from the E-SE direction are relatively high, followed by the NW and NE directions. TY is adjacent to the Beijing–Tianjin–Hebei urban agglomeration in the east, and it is among the “2 + 26” cities, which regional pollution of particulate matter is relatively serious. The high concentration of particulate matter in the Beijing–Tianjin–Hebei region will have an impact on local PM_10_ and PM_2.5_ concentrations after long-distance transmission, which is consistent with the result of the trajectory cluster analysis in the next section.

**Figure 6:**
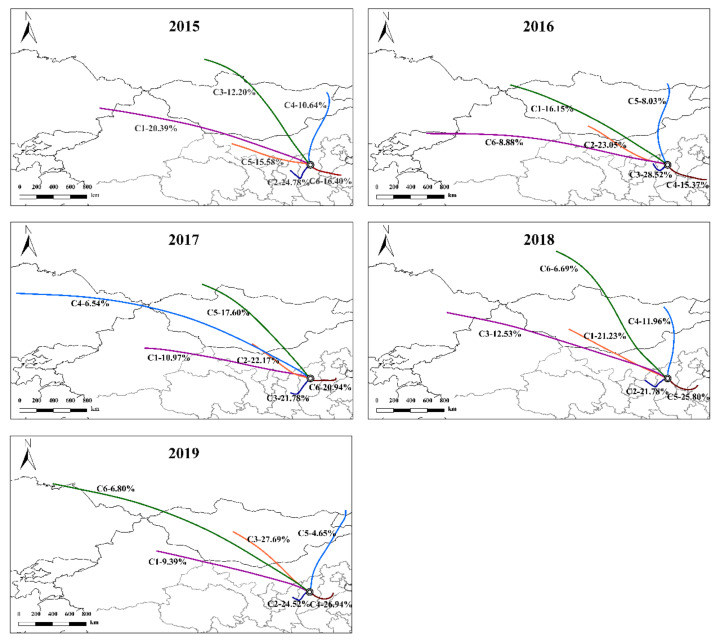
Backward trajectory clustering of Taiyuan city from 2015 to 2019.

In this study, the number of clusters is determined to be 6, and the clustering results are shown in Figure 6. In 2015, trajectory C2 was from the southwest direction, which accounted for the highest proportion 24.78%, and it could be seen from Figure S2 that its PM_2.5_ concentration was the highest. Trajectory C2 was from LF and Yanan city in the southwest direction and had a short moving path, indicating that it was mainly influenced by local source emissions in surrounding cities. Meanwhile, the PM_2.5_/PM_10_ value of C2 also was the highest, indicating that air mass from this direction contributed significantly to local fine particles. Trajectory C3 from the northwest direction and the C4 from the northeast direction both originated from Mongolia, and the PM_10_ and PM_2.5_ concentration were the lowest, which expressed the clean air mass from this district was conducive to the diffusion of particles matter, thus reducing the PM concentration in TY.

Similar to 2015, the trajectory C3 from the southwest direction still accounted for the highest proportion with 28.52% in 2016, and its PM_2.5_/PM_10_ ratio reached the highest among all trajectories. The PM_10_ and PM_2.5_ concentration of trajectory C6 from the western direction was the highest, which originated from Xinjiang.

In 2017, trajectory C2 from the northwest accounted for the highest proportion of 22.17%. The PM concentration of trajectory C1 from the northwest was significantly higher than that of other trajectories, and it originated from central Xinjiang, northern Gansu and western Inner Mongolia, which was the main source of sandstorms in China and transported dust to TY. Multiple sandstorms in 2017 significantly increased the concentration of PM_10_ in the region.

In 2018, the trajectory C5 from the eastern direction had the highest proportion of 25.80% and its PM_2.5_/PM_10_ ratio was the highest. Although the trajectory C3 from the northwest direction accounted for proportion with 12.53%, it had the highest PM_10_ concentration. This trajectory passed through northern Xinjiang and western Inner Mongolia, and its contribution to local PM_10_ concentration increased due to frequent dust weather.

In 2019, the trajectory C3 from Mongolia accounted for the highest proportion with 27.69%, followed by trajectory C4 with 26.94%. However, the PM_2.5_/PM_10_ ratio of trajectory C4 was the highest. It is worth noting that the proportion of air mass from the eastern direction has been increasing gradually, indicating that regional transport from the Beijing–Tianjin–Hebei region has a continuously increasing influence on local PM_10_ and PM_2.5_ concentration.

In general, during 2015–2019, the air mass trajectory of TY is mainly from the northwest direction, followed by the southwest direction and the east direction. In the northwest, there are many long tracks of long-distance transmission each year.

The clustering analysis of the trajectories has determined the transport direction and proportion of the trajectories in Taiyuan. In order to further reveal the spatial distribution characteristics of potential pollution sources in the study area, the contribution factor analysis of the potential source area was carried out for the backward trajectories of PM_2.5_ concentration exceeding the standard in Taiyuan from 2015 to 2019. The threshold concentration is set as the secondary standard limit of Ambient Air Quality Standard (GB3905-2012).

**Figure 7:**
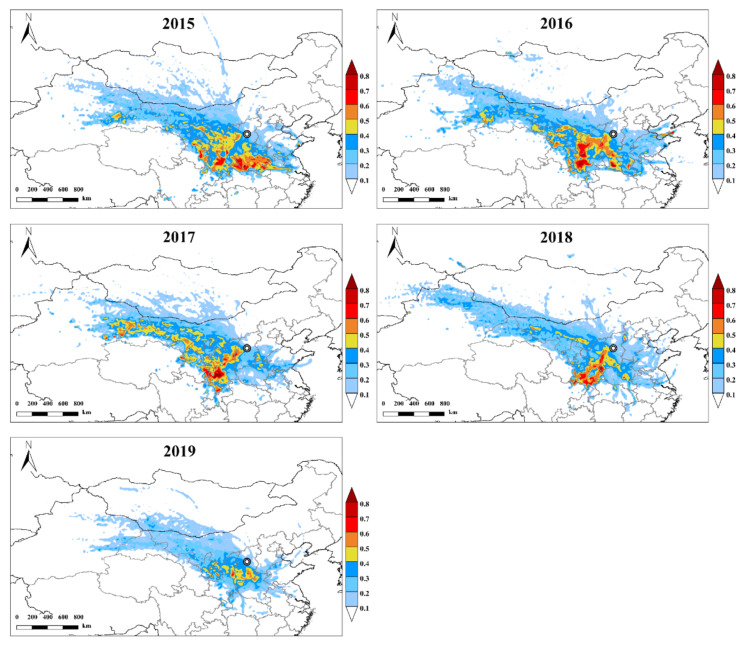
PSCF distribution of PM_2.5_ in Taiyuan from 2015 to 2019.

Figure 7 shows the PSCF distribution of PM_2.5_ in TY from 2015 to 2019. In 2015, the PM_2.5_ level in TY was mainly affected by the southwest, northwest and south directions, with a large range of potential pollution sources. The high WPSCF value was mainly distributed in Henan, northern Anhui, Shaanxi, Ningxia and eastern Gansu, and it exceeded 0.8 in western Henan and southern Shaanxi. In 2016, the value of WPSCF in Henan decreased significantly, and the transport of fine particulate pollution from the region weakened significantly. The high WPSCF values were concentrated in the southwest of Shaanxi province and the east of Gansu Province, which were >0.7. In 2017, the high WPSCF values were still concentrated in southwest Shaanxi province and eastern Gansu Province, but the range decreased. Meanwhile, compared with the previous two years, fine particulate matter transport from central Xinjiang in the northwest direction increased significantly, reaching the highest value in several years (WPSCF value > 0.4). In 2018, the value of WPSCF in Henan increased and its contribution increased significantly. The WPSCF value in 2019 was significantly lower than that in the past few years, and the range was significantly reduced. It is worth noting that, from the central and northern parts of Xinjiang through northern Gansu, western Inner Mongolia, northern Shaanxi and other regions to TY, there is a potential contribution source belt covering a wide range of northwest to southeast every year, which has a great contribution to the mass concentration of PM_2.5_.

In general, the southwest area of TY is a high potential source area of fine particulate matter pollution. This area is the core area of the Fenwei Plain, including Baoji City, Xianyang City, Tongchuan City, Xi’an City and other regions in Shaanxi Province, as well as Lvliang City, Linfen City and Yuncheng City in Shanxi Province. The results show that the Fenwei Plain is the main source of PM_2.5_ in Taiyuan City, which is consistent with the findings of Yan [51] et al. Further, the long-distance transport of PM_2.5_ in the dust source area from the northwest also contributes to local fine particulate matter pollution to a certain extent; therefore, implementing policies such as windbreak and sand fixation, afforestation and mitigation of land desertification, and vigorously carrying out regional joint prevention and control can effectively alleviate the long-distance transmission of PM_2.5_ in Northwest China.

In addition, when the distribution of potential source areas is clarified, the contribution of potential source areas to local air pollution can be preliminarily understood, and the understanding of PM_2.5_ transport routes and potential sources in Taiyuan City can be improved. This can provide a scientific basis for air pollution prevention and control, provide a reference for effectively controlling air pollution in Taiyuan City and carry out regional joint prevention and control, and at the same time help reduce inter-regional human health risks.

## 4. Conclusions

In this study, 12 cities located in Shanxi Province, Inner Mongolia Autonomous Region and Shaanxi Province were selected as the study area to explore the spatial and temporal distribution characteristics of particulate matter in this region, and analyze the effect of social-economic and natural factors on local particulate matter level. The conclusions are as follows:

(1) During 2015 to 2019, the average annual concentration of PM_2.5_ and PM_10_ increased first and then decreased, reaching the peak in 2017. Temporally, PM_2.5_ and PM_10_ presented a “U” shaped change pattern for monthly variation, and a double-peak and double-valley pattern for diurnal variation. Spatially, PM_2.5_ and PM_10_ concentration of TY and YQ in the central region and LF and JC in the southern region is higher, and EEDS and YL in the western region are relatively light.
(2) In terms of social-economic factors, PM_2.5_ has a significant correlation with SI, PD, GC and CO. Differently, PM_10_ has a significant correlation with GC and CO, and a poor correlation with other social-economic factors. Specifically, PM_2.5_ has an “inverted U-shaped” quadratic polynomial relationship with SI and PD, and a U-shaped relationship with GC and CO.
(3) In terms of natural factors, PM_2.5_ and PM_10_ are significantly positively correlated with NO_2_, SO_2_, CO and Pressure, and significantly negatively correlated with O_3_ and Temperature. Notably, wind speed has a significant negative correlation with PM_2.5_ and a significant positive correlation with PM_10_. Moreover, backward trajectory cluster analysis shows that the air mass trajectory in TY is mainly from the northwest direction, followed by the southwest direction and the east direction. In the northwest, there are many trajectories of long-distance transmission each year. PSCF analysis results show that the southwest region of TY is a high potential source of fine particulate matter pollution, and the long-distance transport of PM_2.5_ from Xinjiang in the northwest also contributes to fine particulate matter pollution to a certain extent. This paper discusses the influence of natural factors and social and economic factors on the concentration of particulate matter in coal production cities. In the follow-up work, the influence of various industries on particulate matter pollution can also be detailed, which will help decision-makers to consider these related air pollution conditions when formulating future urban development policies.

## Figures and Tables

**Table 1:** Results of curve fitting.

		PM_2.5_					PM_10_				
Year	Parameter	SI	GDP	PD	GC	OC	SI	GDP	PD	GC	OC
2015	R2	0.64 *	0.24	0.52 *	0.72 **	0.73 **	0.37	0.10122	0.73 **	0.45	0.53 *
	b1	0.05570	0.00077	0.14981	0.04629	−0.00079	0.05915	−0.01826	0.19445	0.03317	−0.00076
	b2	−0.00004	0.00000	−0.00017	−0.00011	0.00000	−0.00004	0.00000	−0.00020	−0.00009	0.00000
	Constant	37.31681	55.43423	29.56998	50.60329	61.89346	79.40285	111.18117	65.78631	96.47074	105.85320
2016	R2	0.66 **	0.24	0.57 *	0.66 **	0.63 *	0.52 *	0.20	0.65 **	0.66 **	0.70 **
	b1	0.08242	0.01819	0.22010	−0.00564	−0.00102	0.07577	−0.02957	0.29224	−0.10892	−0.00203
	b2	−0.00005	−0.00001	−0.00025	−0.00006	0.00000	−0.00005	0.00001	−0.00032	0.00003	0.00000
	Constant	30.40025	46.04485	21.45023	63.14374	65.60328	83.15594	129.85351	58.40048	131.28330	122.99882
2017	R2	0.53 *	0.24	0.48	0.72 **	0.72 **	0.50 *	0.17	0.44	0.76 **	0.71 **
	b1	0.04309	0.00037	0.21455	−0.06208	−0.00156	0.06639	−0.02285	0.22438	−0.14540	−0.00244
	b2	−0.00003	0.00000	−0.00025	0.00000	0.00000	−0.00004	0.00000	−0.00022	0.00007	0.00000
	Constant	45.00104	62.09259	24.99976	76.73892	73.23390	89.05378	133.89126	73.89322	147.32929	133.74660
2018	R2	0.41 **	0.20	0.56 *	0.79 **	0.71 **	0.30	0.09	0.48	0.55 *	0.55 *
	b1	0.01957	−0.01212	0.17716	−0.07201	−0.00126	0.02991	−0.02713	0.11934	−0.06933	−0.00163
	b2	−0.00001	0.00000	−0.00020	0.00003	0.00000	−0.00001	0.00001	−0.00008	0.00002	0.00000
	Constant	46.30296	65.98086	24.57951	72.64170	64.69729	99.06534	135.54831	87.24706	130.11946	125.60507
2019	R2	0.25	0.10	0.57 *	0.64 *	0.60 *	0.36	0.18	0.32	0.55 *	0.54 *
	b1	0.00934	−0.00741	0.14579	−0.08980	−0.00090	0.01014	−0.01464	0.14274	0.02075	−0.00069
	b2	−0.00001	0.00000	−0.00015	0.00005	0.00000	−0.00001	0.00000	−0.00014	−0.00004	0.00000
	Constant	43.18883	53.97019	22.01969	70.07539	55.57668	92.72066	110.43906	69.47818	94.19326	102.71590

** At level 0.01, the correlation is significant. * At level 0.05, the correlation is significant.

**Table 2:** Results of Pearson correlation coefficient.

	PM_2.5_	PM_10_	SO_2_	NO_2_	O_3_	CO	T	P	WS
PM_2.5_	1								
PM_10_	0.869 **	1							
SO_2_	0.713 **	0.644 **	1						
NO_2_	0.599 **	0.584 **	0.559 **	1					
O_3_	−0.307 **	−0.317 **	−0.319 **	−0.679 **	1				
CO	0.873 **	0.713 **	0.789 **	0.656 **	−0.355 **	1			
T	−0.352 **	−0.333 **	−0.445 **	−0.258 **	0.408 **	−0.344 **	1		
P	0.275 **	0.261 **	0.365 **	0.266 **	−0.544 **	0.306 **	−0.862 **	1	
WS	−0.079 **	0.042 *	−0.093 **	−0.123 **	0.040 *	−0.132 **	0.137 **	−0.074 **	1

** At level 0.01, the correlation is significant. * At level 0.05, the correlation is significant.

## Data Availability

Publicly available datasets were analyzed in this study. The data sources are https://www.aqistudy.cn/historydata/(accessed on 21 September 2021) and http://data.cma.cn (accessed on 25 September 2021).

## Supplementary Materials

The following supporting information can be downloaded at: https://www.mdpi.com/article/10.3390/ijerph19063228/s1, Figure S1: 2015–2019 Annual average concentration of particulate matter in cities (a) PM2.5 (b) PM10; Figure S2: 2015–2019 city socioeconomic indicators SI, GDP, PD, GC left axis, CO right axis; Figure S3: Curve fitting diagram of particulate matter concentration and socioeconomic factors in 2015 and 2016; Figure S4: Curve fitting diagram of particulate matter concentration and socioeconomic factors in 2017 and 2018; Figure S5: Curve fitting diagram of particulate matter concentration and socioeconomic factors in 2019; Figure S6: Statistical chart of 6 types of trajectories PM2.5, PM10, PM2.5/PM10 from 2015 to 2019 (one row for one year); Table S1: Socioeconomic Factor Indicators.
