# Supplementary material for: Study on the Spatial and Temporal Distribution Characteristics and Influencing Factors of Particulate Matter Pollution in Coal Production Cities in China
Source: Int J Environ Res Public Health. 2022 Mar 9;19(6):3228. doi: 10.3390/ijerph19063228 (PMC8950844; doi:10.3390/ijerph19063228)
Supplement: Supplementary file 1 [file ijerph-19-03228-s001.zip › Supplementary File.pdf]

International Journal of
Environmental Research and Public Health

MDPI

Article

Study on the Spatial and Temporal Distribution Characteristics and Influencing Factors of Particulate Matter Pollution in Coal Production Cities in China

Ju Wang *, Tongnan Li, Zhuoqiong Li and Chunsheng Fang

College of New Energy and Environment, Jilin University, Changchun 130012, China; litn20@mails.jlu.edu.cn (L.T.); zhuoqiong21@mails.jlu.edu.cn (L.Z.); fangcs@jlu.edu.cn (F.C.)
* Correspondence: wangju@jlu.edu.cn; Tel.: +86-131-0431-7228

Citation: Wang, J.; Li, T.; Li, Z.; Fang, C. Study on the Spatial and Temporal Distribution Characteristics and Influencing Factors of Particulate Matter Pollution in Coal Production Cities in China. *Int. J. Environ. Res. Public Health* **2022**, *19*, 3228. https://doi.org/10.3390/ijerph19063228

Academic Editor: Paul B. Tchounwou

Received: 8 February 2022
Accepted: 8 March 2022
Published: 9 March 2022

Publisher's Note: MDPI stays neutral with regard to jurisdictional claims in published maps and institutional affiliations.

Copyright: © 2022 by the author. Licensee MDPI, Basel, Switzerland. This article is an open access article distributed under the terms and conditions of the Creative Commons Attribution (CC BY) license (https://creativecommons.org/licenses/by/4.0/).

Table S1. Socioeconomic Factor Indicators.

Socioeconomic Factors	Abbreviation	Units
Second Industry	SI	10^8 yuan
Gross Domestic Product	GDP	10^8 yuan
Population Density	PD	People/km^2
Generating Capacity	GC	10^4 tons
Coal Output	CO	10^8 KW·H

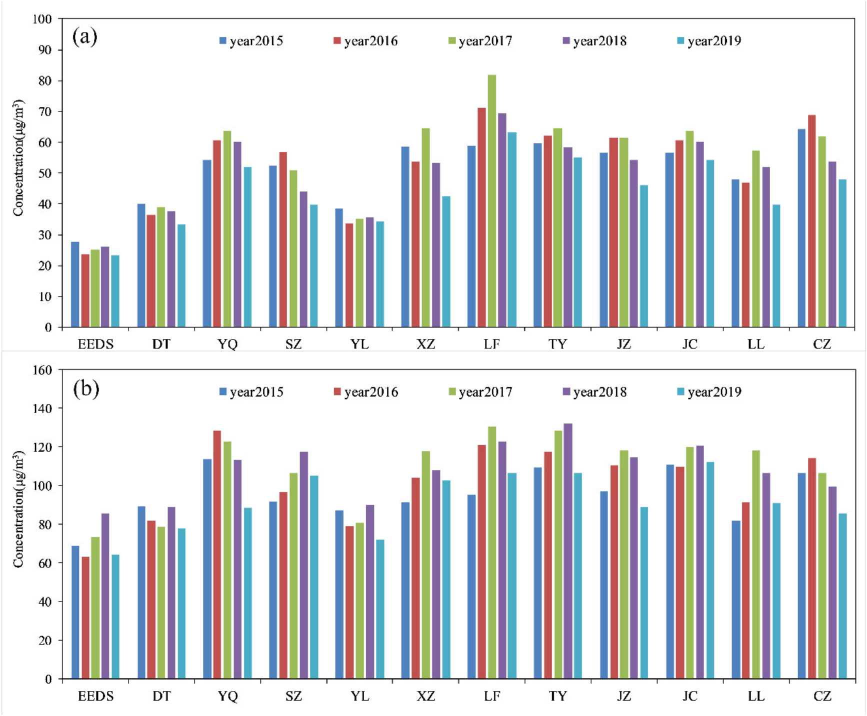

Figure S1. 2015-2019 Annual average concentration of particulate matter in cities (a) $PM_{2.5}$ (b) PM_{10}.

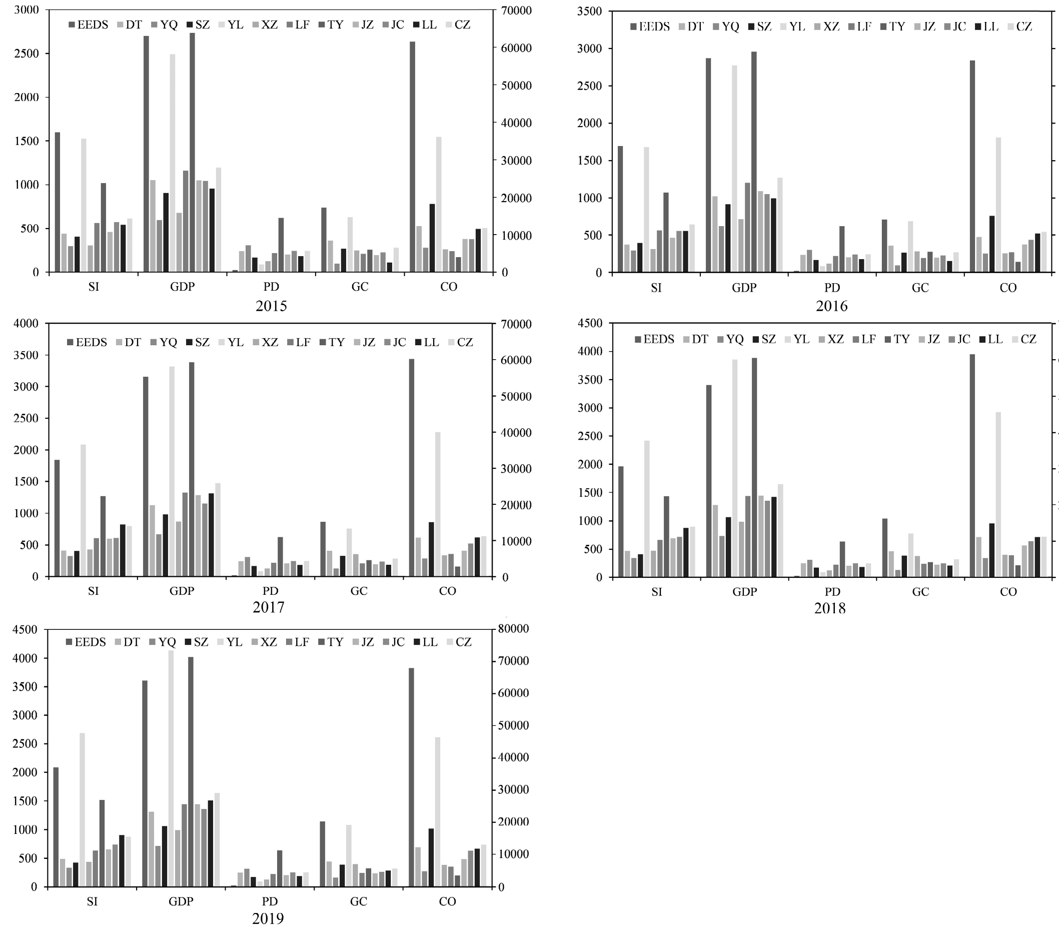

Figure S2. 2015-2019 city socioeconomic indicators SI, GDP, PD, GC left axis, CO right axis.

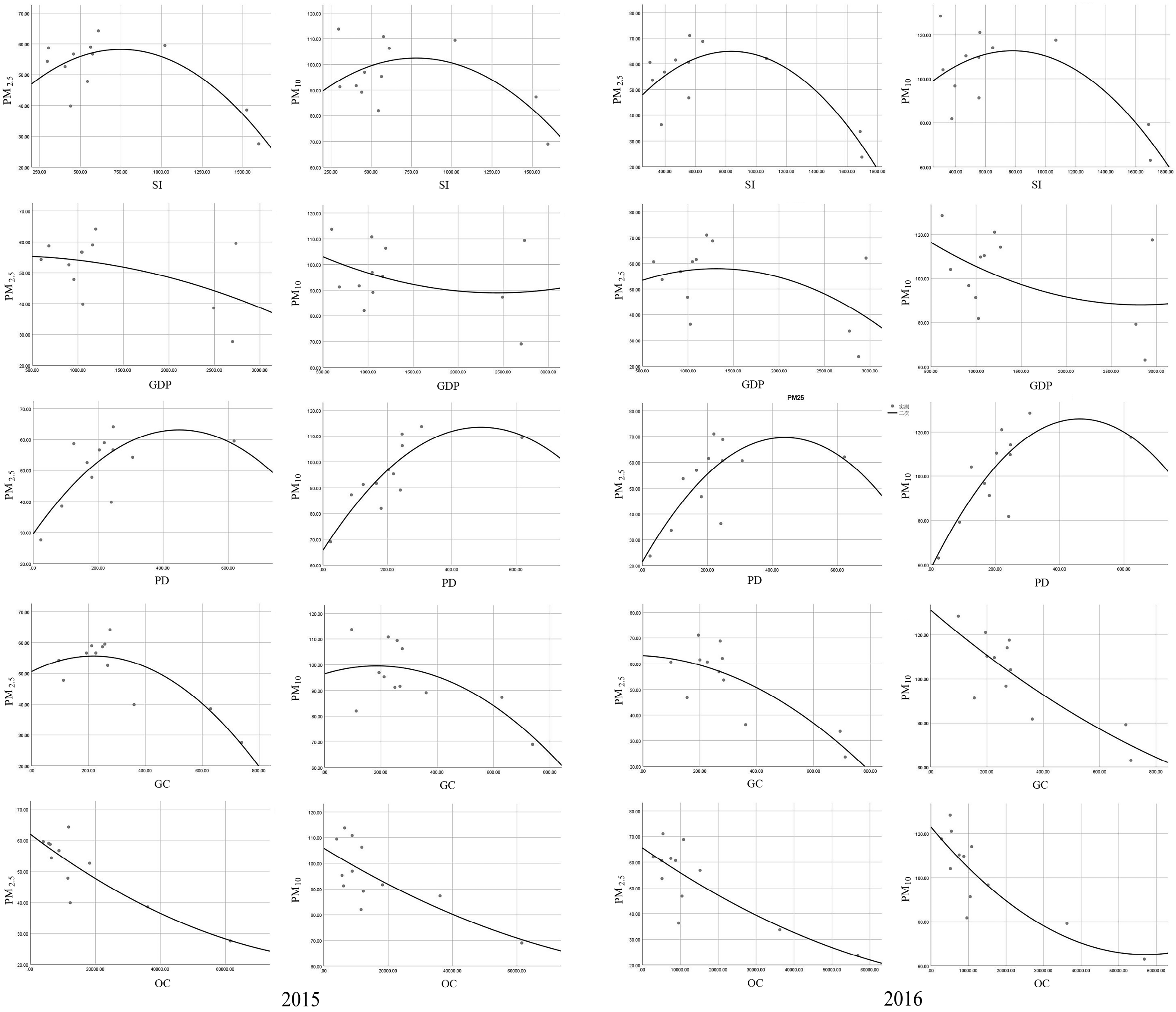

Figure S3. Curve fitting diagram of particulate matter concentration and socioeconomic factors in 2015 and 2016.

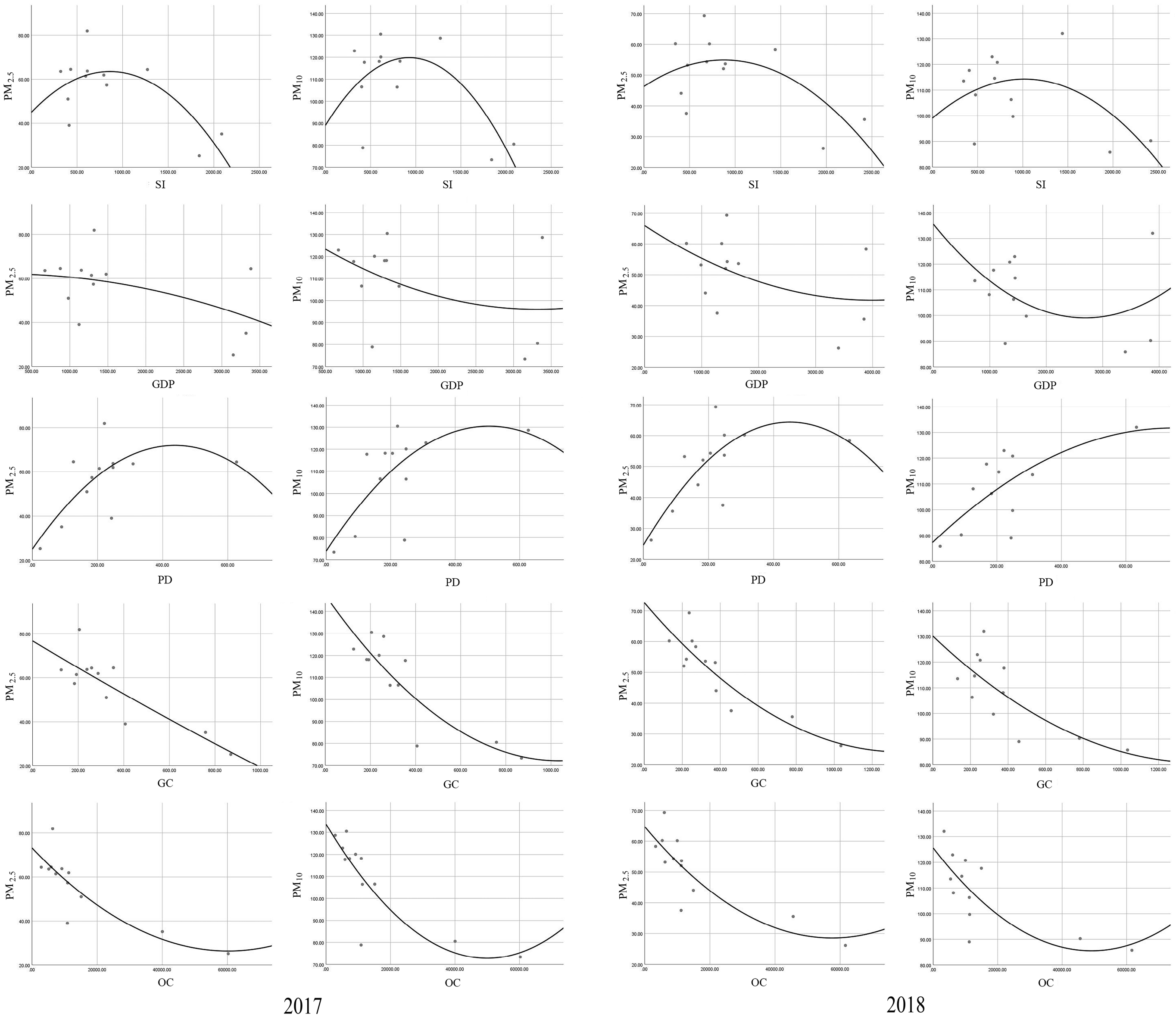

Figure S4. Curve fitting diagram of particulate matter concentration and socioeconomic factors in 2017 and 2018.

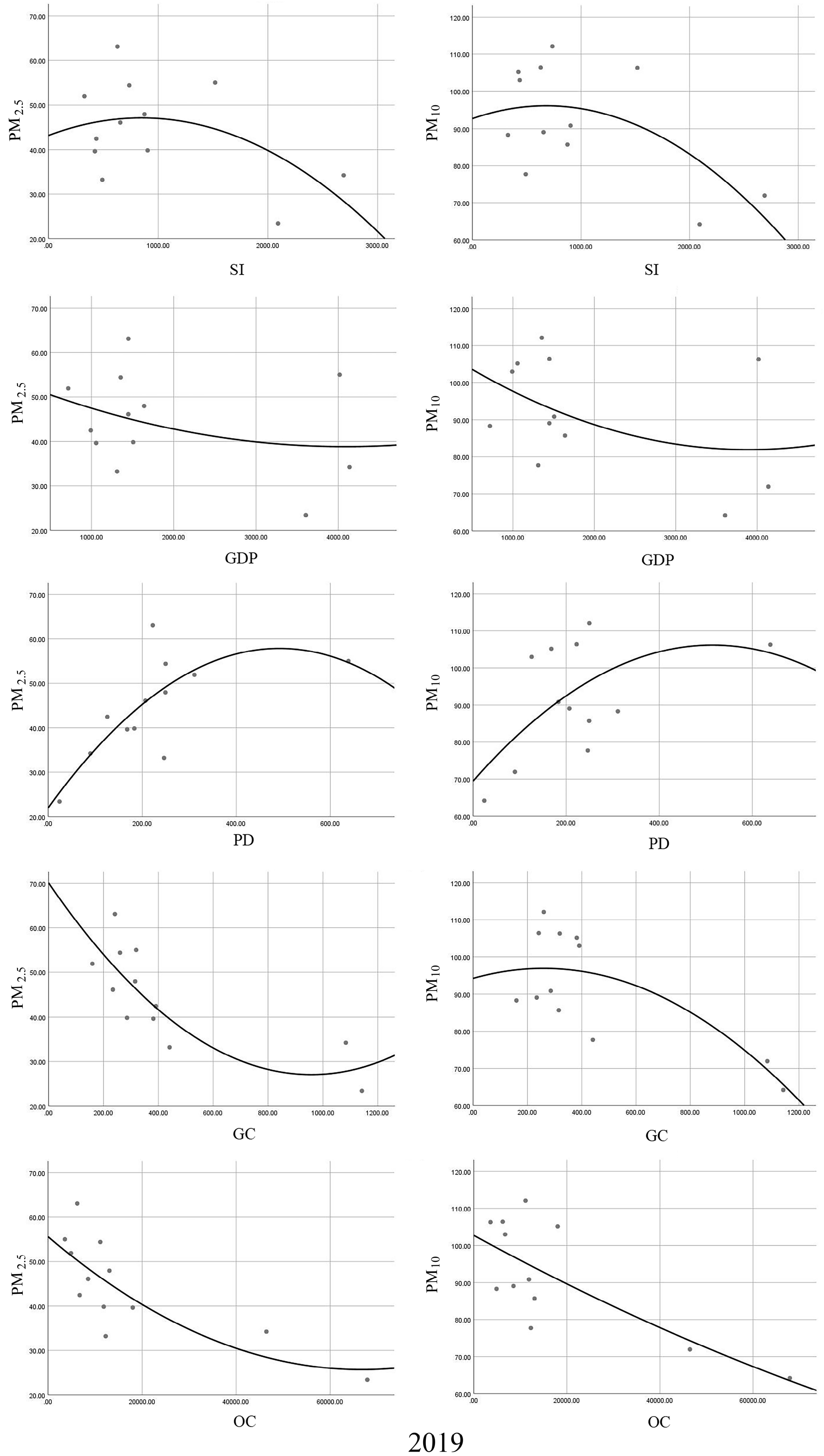

2019

Figure S5. Curve fitting diagram of particulate matter concentration and socioeconomic factors in 2019.

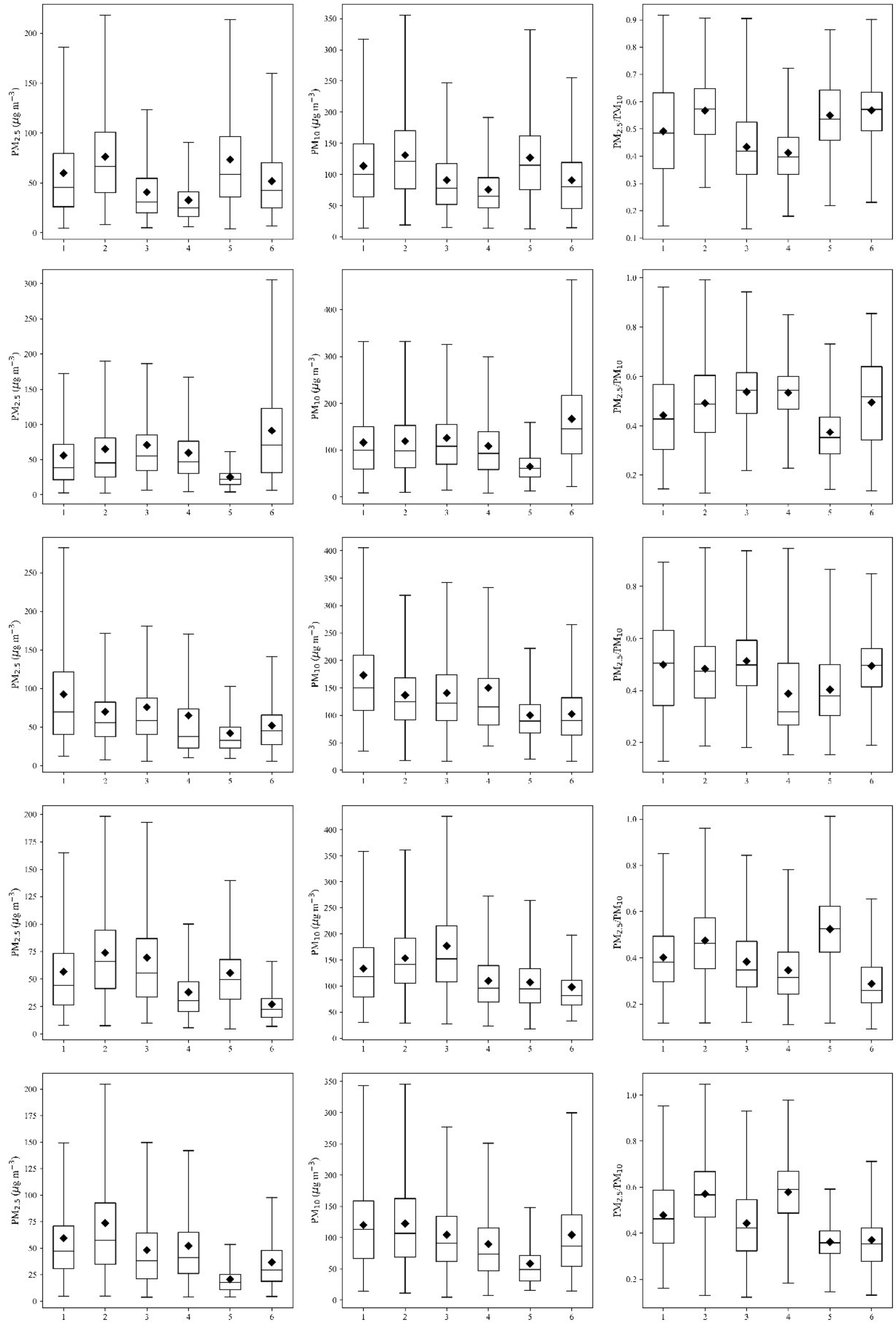

Figure S6. Statistical chart of 6 types of trajectories $PM_{2.5}$, PM_{10}, $PM_{2.5}/PM_{10}$ from 2015 to 2019 (one row for one year).
